# Living with a person with young onset dementia – spousal experience

**DOI:** 10.1080/17482631.2024.2330233

**Published:** 2024-03-17

**Authors:** Tania Håkansson, Hilda Svensson, Staffan Karlsson

**Affiliations:** a School of Health and Welfare, Halmstad University, Halmstad, Sweden; b Faculty of Health Science, Kristianstad University, Kristianstad, Sweden

**Keywords:** Young onset dementia, everyday life, spouse, experience, emotional impact

## Abstract

**Purpose:**

Being of working age while at the same time needing to help a partner with young onset dementia has specific consequences for spouses. Research to date has been sparse concerning this particular group of spouses. The aim of the study was to explore spouses’ everyday experiences when living with a person with young onset dementia.

**Method:**

The study had a descriptive qualitative design with semi-structured interviews with nine spouses. The interviews were analysed using content analysis.

**Result:**

The interviewed spouses experienced emotions that varied from feelings of loneliness, frustration, and worry to peace of mind. They said that they used coping strategies, which included adopting a positive mindset, adapting to inabilities, adopting an avoidant approach, and finding ways to recharge. Spouses also felt that they could use more support, both formal and informal.

**Conclusion:**

The spouse of a person with young onset dementia has a range of emotional experiences and has resourceful ways of handling everyday life. Various types of support are offered to spouses, however, they seemed to desire more from health care services.

## Introduction

Today 55 million people worldwide are living with dementia, and one person is diagnosed with dementia every three seconds; accordingly, the World Health Organization (WHO) acknowledges dementia to be a public health priority (Alzheimer’s Disease International ADI, 2014; World Health Organization, 2022). Although it is usually thought of as a disease of older people, dementia can strike people under the age of 65, in which case it is called young onset dementia (World Health Organization, 2023). Young onset dementia is estimated to affect 119 per 100,000 people, which means that there are 3.9 million people living with young onset dementia globally (Hendriks et al., 2021). In Sweden, approximately 130,000–150,000 people have dementia (The National Board of Health and Welfare, 2017), it is estimated that two to 10% are under the age of 65 of those with dementia (World health organization, 2012).

There are several types of dementia, but the most common is Alzheimer’s disease (World Health Organization, 2023) which accounts for about 15–40% of people with young onset dementia (Draper & Withall, 2015). Alzheimer’s is a degenerative illness caused by cell death in the brain. Typical early symptoms of Alzheimer’s can be difficulty remembering recent events, problems with orienting oneself in familiar places, and language dysfunction (World Health Organization, 2023). However, a previous study by Koedam et al. (2010) showed that a third of participants with young onset dementia showed no signs of memory impairment. The period between first symptoms and diagnosis was estimated to be approximately 3.5 years for people with young onset dementia (Koedam et al., 2010), which is longer than for those with dementia over the age of 65 and could be attributed to the fact that even professionals see dementia as a disease of older people, and that symptoms for individuals with young onset dementia are atypical (Mendez, 2006). Another reason could be that people with young onset dementia more frequently get a psychiatric diagnosis before being diagnosed with a dementia (Johannessen & Möller, 2011; Koedam et al., 2010).

Depending on the stage of the illness, people with dementia rely on support from family caregivers to function and have a good quality of life while still living at home (Brodaty & Donkin, 2009; The National Board of Health and Welfare, 2017). According to World Alzheimer Report (2022), 54% of family caregivers experience feeling stressed due to their role as a caregiver. Being of working age while at the same time needing to help a person with young onset dementia has additional consequences for a spouse (Holthe et al., 2017; Roach & Drummond, 2014; Vliet et al., 2010). Almost 60% of family caregivers have said they spent more than five hours a day supervising or helping the person with young onset dementia (Stamou et al., 2020). A study in France found that spouses of persons with young onset dementia have described that they have had to take on domestic responsibilities that they had not been accustomed to doing (Wawrziczny et al., 2016); a similar study in UK found 40% of family caregivers reported spending about 15 hours a week on household tasks that they had not performed previously (Stamou et al., 2020). Sweden’s The National Board of Health and Welfare (2017) reports that family caregivers often neglect their own needs to the extent that they themselves become ill, either physically or mentally. Furthermore, spouses of people with young onset dementia have described how being the soul provider of the family after the spouse had fallen ill, had become burdensome along with the added burden of household tasks (Binford et al., 2023). According to the Swedish Social Services Act (SFS 2009:549), Swedish municipalities are obligated to provide support to family caregivers who are caring for a person that has a long-term illness, is older, and/or is disabled. The support provided by municipalities needs to be flexible, individual, and of a quality that fits the needs of the family caregiver (The National Board of Health and Welfare, 2021). However, specific daily activity centres for people with young onset dementia are available in fewer than a fifth of municipalities in Sweden (The National Board of Health and Welfare, 2017).

With their many areas of responsibility, respite is a necessity for family caregivers (Wawrziczny et al., 2017). Research shows that 70% of family members of people with young onset dementia reported having no regular respite, with approximately 45% reporting needing help from friends and family to manage (Stamou et al., 2020). Kilty et al. (2023) stated that families of people with young onset dementia often were left unsupported without knowing who to turn to regarding retirement funds and employment rights. The loss of income from the person with dementia not being employed after diagnosis had left the spouses forced to take on employment while supporting the person with dementia. To date, finding age-appropriate services for people with young onset dementia is challenging to say the least (Roach & Drummond, 2014; Wawrziczny et al., 2017). In addition, almost half of family caregivers have reported having no follow-up six weeks post-diagnosis for the person with young onset dementia (Stamou et al., 2020). Furthermore, 70% of family caregivers reported not having attended a support group, nor had they any other organization provide care for the person with young onset dementia (Stamou et al., 2020).

Spouses of persons with young onset dementia are usually still active in the workforce, in the middle of life having adult children, grandchildren and older parents to consider. Their unique situation of living with a person who has dementia is underrepresented in research. Understanding their lived experience of everyday life is central for a better understanding of their needs and development of aimed support. Clearly, further research is needed.

## Aim

The aim of the study was to explore spouses’ experiences of everyday life when living with a person with young onset dementia.

## Materials and methods

### Design

The study had a qualitative descriptive design based on semi-structured interviews with spouses of persons with young onset dementia, followed by inductive content analysis. The aim of the study determines the method (Doyle et al., 2020) and an inductive qualitative approach is appropriate when analysing peoples lived experiences (Granheim & Lundman, 2004; Granheim et al., 2017). Qualitative research wants to understand the lived experience of people, which requires a flexibility when analysed without losing the essence of the phenomenon that is being studied. Qualitative research is concerned with the who, what and where of peoples lived experience (Doyle et al., 2020). This study explored spouses’ experiences of everyday life when living with a person with young onset dementia.

### Recruitment and participants

Nine spouses were included in the study (Table 1). Criteria of inclusions were independence in activities of daily living (ADL) and their living in their own home with a person with young onset dementia (<20 points MMSE). Exclusion criteria were not being able to communicate in Swedish. The score in an S-MMSE test reflects the cognitive ability of the person with dementia (Molloy et al., 1991) which is of importance for describing the context of the situation that the spouses are living in. The first author performed the S-MMSE test in the privacy of the person with dementia’s home. To protect the integrity of the person with dementia, S-MMSE scores were not conveyed to their spouse. The first author received permission from head of department at one municipality and five memory clinics in the Southwest of Sweden, to contact dementia nurses in their organization. The spouses were identified due to the persons with young onset dementia being patients at the municipalities and memory clinics. The municipalities and the memory clinics conveyed contact information of the spouses to the researchers after obtaining permission from those spouses. The spouses were contacted by phone so that the first author could describe the aim of the study, what participation would involve, and answer potential questions as well as being informed of their right to withdraw their participation at any time. All participants conveyed agreed to participate in the study.

**Table 1.** Participant demographics, *n* = 9.

Spouse	
Age, mean (range)	59(52–64)
Women, n	6
Heterosexual orientation	8
Has Children	7
Children residing at home	0
Education, n	
University level	3
Senior high school	6
Setting, n	
Urban	7
Rural	2
Cohabitating person with dementia MMSE score, mean (range)	26 (20–29)
Age person with dementia, mean (range)	60 (58–64)

### Data collection

The data was collected from September 2020 to March 2022. After verbal consent to participate, an appointment for interview was set up in the privacy of the spouse’s home. As the participants and the first author had received the vaccine for the Covid −19 virus, the spouses felt comfortable with being interviewed in person. A letter of information and a consent form were sent to the spouses to read and sign at their own convenience. The consent form was collected before the interview. The interviews were not performed in the presence of the person with young onset dementia. Socio-demographic data was collected (Table 1). The first author performed all interviews in the privacy of the spouse’s home, using a digital recording. The spouse chose where in the home the interviews took place, which was mainly at the kitchen table. The first author has a background of psychiatric nursing and has experience approaching people with dementia regarding support and care. According to Lester and O’Reilly (2021) the quality of the interaction of the participant and the researcher can strengthen the validity of the data. The main research question asked in the interviews was: “How would you describe everyday life since your spouse has received the diagnosis?” If the spouse had trouble getting started, the first author used follow-up questions to get the interview going, such as “can you describe how everything started?” or “what challenges did you face in everyday life?” The semi structured interview guide assisted the interviews to remain within the same area but also left space for the spouses to speak freely. The interviews ranged from 23 minutes to 110 minutes, with an average of 63 minutes. The interviews were transcribed verbatim by the first author.

### Data analysis

For description of spouses’ experiences, a qualitative content analysis with an inductive approach according to Granheim and Lundman (2004) and Granheim et al. (2017) was used to analyse the interviews. The first author read the transcribed interviews several times to get an understanding of the whole, the interviews were discussed with the co-authors. The text that responded to the aim was extracted forming meaning units. The meaning units were then condensed, close to the text (manifest), the condensed meaning units were then interpreted (latent). The condensed meaning units were then abstracted into sub-themes. The sub-themes were then formed into themes (Table 2) (Granheim & Lundman, 2004; Granheim et al., 2017).

**Table 2.** Examples of analytical process.

Meaning unit	Condensed meaning unit Description close to the text	Condensed meaning unit Interpretation of the underlying meaning	Sub-theme	Theme
”He hears me, but he can’t really understand and reason the way that he did before. He would have given me some guidance or asked questions so that the discussion or my thoughts would have evolved. Yes, it’s very sad because I feel like I have lost my most significant conversational partner.”	Before he would have given me some guidance or asked questions. It’s very sad because I feel like I have lost my most significant conversational partner.	Grieving the loss of my most valuable sounding board	Loneliness in a dyad	Experiencing an emotional impact
“But mainly I feel great working out as well. So, I work out three, four times a week. And it doesn’t take too much time. I go on those 45 min workouts. I go to those trainer-lead exercises. Group training and such, then you boost your serotonin levels and its relaxing and its, it’s really great to sometimes be able to get out the house in the evening.”	I feel great working out, so I work out three or four times a week. I go to those trainer lead exercises. It boosts your serotonin levels, and it is relaxing to be able to get out the house in the evening.	Focusing on my own well-being	Finding ways to recharge	Using coping strategies

### Ethical consideration

The interviews could evoke difficult thoughts and feelings in the spouses, but the importance of understanding the spouse’s everyday life experiences outweighed the potential for spouses to be temporarily saddened during interviews. The first author, the memory clinics and municipalities had agreed to support the spouses should they need it after being interviewed. The letter of information told the spouses that they would be confidential and gave them a thorough understanding of their rights to withdraw consent at any time. The spouses were asked to not have the interviews in the presence of the person with dementia so that they would feel free to express their lived experiences. However, the person with dementia was still in the house or apartment during the interview, but in a different room with the door closed. The risk of interviewing the spouses at home where the person with dementia was weighted against the benefit of the spouses being in a natural environment in which they felt comfortable. The research was approved by the Swedish Ethical Review Authority (Dnr. 2020–00571).

## Results

Analysing the data revealed 10 subthemes and three main themes (Table 3). The spouses reported experiencing emotions that varied from feelings of loneliness, frustration, and increased worry to having found peace of mind. Spouses described using coping strategies, which revealed the resourceful ways in which they handled everyday life. These strategies included adopting a positive mindset, adapting to inabilities, adopting an avoidant approach, and finding ways to recharge. The spouses also called for increased support, including both informal and formal support. The Appendix shows the frequency of the experiences found in the interviews. The spouses are considered a couple (two), a few (three), some (four to six) and most (seven to eight) in the results.

**Table 3.** Results with themes and sub-themes.

Themes	Sub-themes
Experiencing an emotional impact	Loneliness in a dyad
	Feelings of frustration
	Increasingly worried
	Peace of mind
Using coping strategies	Adopting a positive mindset
	Adapting to inabilities
	Adopting an avoidant approach
	Finding ways to recharge
Calling for support	Informal support
	Formal support

### Experiencing an emotional impact

A wide range of emotions were experienced in the everyday lives of people with a spouse with young onset dementia. Most spouses (seven to eight) said that the mental exchange was decreased and was not as it had been before (for example, before dementia, they may have talked about how to handle difficulties at work). Not having that same mental exchange brought on feelings of loneliness. Even though they expressed feelings of frustration, some spouses (four to six) experienced having peace of mind in knowing that the person with young onset dementia had a meaningful everyday life.

#### Loneliness in a dyad

Some spouses (four to six) said that they had stopped consulting with the person with young onset dementia, as the responses they received were no longer on the same level as before. They experienced that the issues that they would want to discuss were too complex for the person with young onset dementia to understand. Not being able to discuss issues at a deeper level brought a sense of not being on the same wavelength anymore, and feelings of estrangement. Some spouses (four to six) also said they felt estranged when the person with young onset dementia was unable to engage in issues regarding the family. Most spouses (seven to eight) expressed that conversations were repetitive. A few spouses (three) also felt that the person with young onset dementia was unempathetic with what was happening in their joint lives.

… he’s quite changed in his personality. He … I don’t get as much mental stimulus from him anymore. You are not really on the same wavelength anymore. His world of interest has become a little smaller, and he is a little less aware or interested or updated about certain things. Interview 4

Some spouses (four to six) expressed feelings of estrangement and not being able to interact as previously, which left them feeling lonely and solely responsible for decisions. They experienced that they were left alone with their own problems. A few spouses (three) expressed feelings of hopelessness, not knowing when life would get better. They said that much of everyday life was routine: going to work, coming home, and preparing dinner. New or interesting experiences like travelling did not happen as frequently anymore because they were too strenuous. Not being able to do things they had previously enjoyed doing together made the spouses feel confined. Some spouses (four to six) said that they realized that they had had an unrealistic view of what life would be after they were both retired and were taken aback by how quickly the illness progressed, which meant that the hopes and dreams they had had would not come to be. Most spouses (seven to eight) described a loss of hopes and dreams, which made them feel sad, for it was not the future they had envisioned.

This isn’t what I had envisioned. We were going to grow old together and become … I mean we are still of working age, and we were supposed to enjoy retirement and travel and all of that. We had talked about moving to Spain. Interview 2

#### Feelings of frustration

Most spouses (seven to eight) expressed feelings of frustration due to the changed behaviour or inabilities of the person with young onset dementia. Even though the spouses understood that these limitations were caused by an illness, they couldn’t avoid feeling frustrated at times. A few spouses (three) also expressed frustration about not understanding the illness, for instance when the person with young onset dementia could remember details from 40 years ago but forget everyday events. Some spouses (four to six) also felt frustration with themselves for not being able to handle certain situations better.

So those conflicts can arise, they’re stupid, I know that. It’s not like he will fix the door just because I nag him, rather, he might not even take care of the dishes that day. So, I mean it is unnecessary, completely emotional. Interview 4

The spouses expressed frustration with the diminished responses they received from the person with young onset dementia. One spouse experienced that this lessened response made the person with young onset dementia duller than before.

But I told him, my God, I could I have just as well grabbed anyone in the woods and gone for a walk, and I would have had about the same exchange, it’s like a stranger that I have never met. You can’t just drop empty words, that’s really boring. Interview 9

There was irritation related to not being able to have alone time at home. Some spouses (four to six) said that if they ever were to be alone, they had to leave home. Not being able to rely on digital aids was another source of frustration expressed by the spouses. One of the spouses mentioned that they had been used to sharing digital calendars with their spouse; however, the spouse with dementia sometimes deleted events, which rendered the digital aid unreliable. Most spouses (seven to eight) expressed frustration with often having to help look for keys and wallets, as well as helping to answer text messages and emails (which they didn’t used to do). Some spouses (four to six) also said that it felt burdensome to always have to be one step ahead to prevent mishaps, and to not have the same independence from each other anymore. Other sources of frustration included having to be responsible for maintaining social contact, having sole parental responsibility for their adult children, and for having to be responsible for all household tasks.

It’s the whole situation since I have to do everything. He doesn’t have the energy. It’s draining to keep it going somehow. A home needs to be looked after and everything that goes with it. It is weary and I understand, he is tired and all that. Interview 3

#### Increasingly worried

Various sources of worry were named by the spouses, two of the spouses were concerned that the spouse with dementia might get lost while out. A few of the spouses (three) felt worried that there was a chance of their children inheriting the illness. Some spouses (four to six) expressed anxiety about completing important tasks (like making sure legal issues, like getting power of attorney) before it was too late, but also that, when they tried to deal with such issues, anxiety prevented them. One of the spouses were concerned about who would support the person with young onset dementia if something were to happen to him.

Yes, well suppose something happens to me, and my wife needs power of attorney to handle my affairs, but what happens if my wife is further along in her illness? Interview 1

A few spouses (three) were also worried about not receiving support in time or concerned that the support would not be appropriate. Some spouses (four to six) said that they themselves wouldn’t want to be the one providing care for the person with young onset dementia.

And I have said that I don’t want to be the one that takes care of him, I am going to take all the help I can get, I will. I am completely certain of that; I don’t want to be a nurse here at home. Interview 5

#### Peace of mind

Most spouses (seven to eight) described feeling at ease if the person with young onset dementia had a meaningful everyday life. This was especially calming for the spouses who were still working and weren’t at home during the day. Some spouses (four to six) had arranged for meaningful activities themselves, for the person with young onset dementia, which entailed for instance getting a pet to keep the person with young onset dementia company, helping at the local tennis club, or having groceries delivered along with recipes. For the two spouses that had had conversations regarding the future with the person with dementia, expressed feeling at peace.

… to me, I feel relieved knowing that we have discussed it [the future] and that we have, like when you get your papers in order. If something were to happen to either one of us, I would want this. You need to know the thoughts and wishes of the other person. Interview 4

### Using coping strategies

The spouses mentioned various ways that they had adapted to everyday life with a person with young onset dementia, saying for example that they tried to focus on what still worked in everyday life with a positive mindset. The spouses applied an avoidant approach due to the uncertainty of the future, which made the spouses focus more on the present. Another coping strategy mentioned was having found ways to recharge.

#### Adopting a positive mindset

Most spouses (seven to eight) stated that if they focused on what worked instead of what had been lost, life was almost like normal. It was mainly the practical things that were not as before. Although a few had experience of Alzheimer’s in the family, there was still the perception that life could be pleasant. The spouses said that something good had come out of the situation, namely that the focus was more on staying in the moment with the person with young onset dementia rather than tending to responsibilities. Some spouses (four to six) said that they would stay a bit longer in conversation with the person with young onset dementia rather than, for instance, tend to things that needed to be fixed around the house.

Yes, I reprioritize a lot now, it’s much more important yes. When I work from home, we can end up spending one or two hours drinking our morning coffee. If we are deep in conversation, well then, we just continue, the things out there can just wait. Interview 1

A few spouses (three) said that taking over household tasks like cooking was something they didn’t contemplate too much, as it had been happening gradually, and sometimes the tasks were things they realized they enjoyed doing.

#### Adapting to inabilities

Most spouses (seven to eight) had come realize that stress had a negative effect on the abilities of the person with young onset dementia, so they tried to minimize stress by doing things like staying home more often than they might have done otherwise. All of the spouses expressed that they tried to avoid getting into arguments with the person with dementia. For example, if an instruction had gone wrong there was no point in commenting on it.

’You could have just taken that one, why did you bring all three bags?’ There’s like, no point in even saying that you know, so I just said, “oh so you brought all the bags,” “Yeah, I didn’t really know.” “No but that’s fine, I’ll take this one.” I mean you avoid all those arguments that you otherwise would have had. Interview 6

Even though there was no ill intention from the spouses, they said that there needed to be a moment of enlightenment to understand that the person with young onset dementia was unable to change, and that they would have to change their own expectations instead in order to avoid arguments.

Because he doesn’t have the ability to change, that’s where I had to step up and change my expectations … Interview 4

Most spouses (seven to eight) said that there was a fine line between wanting to be supportive and not wanting to intrude upon the integrity of the person with young onset dementia. Some spouses (four to six) had realized that giving little clues in conversations could help the person with young onset dementia find their way back into a conversation. One spouse said that treating every question as if it was new was a good solution for avoiding arguments and not infringing on the autonomy of the person with young onset dementia. Another adaptation was accepting that the person with young onset dementia needed to take powernaps or have downtime at some point during the day.

#### Adopting an avoidant approach

Some spouses (four to six) had an avoidant approach, whether it was about having difficult conversations regarding the future or accepting information regarding the illness. Thinking about the future was subject the spouses were most anxious about. Although spouses stated that they understood that the illness is progressive, and that things would eventually change, the uncertainty of when and how brought on a sense of not wanting to think too much about it for the time being.

I don’t have a clue if my wife will live a normal life for one year or 10 years. Nobody has the answer to that. That’s why there is no point of dwelling on it. It’s better to focus on having a pleasant time right now. Interview 1

Some spouses (four to six) said that that they had found meaningful activities away from home, which they might not have done if the interaction had been more satisfying with the person with young onset dementia.

Still, I’ve filled my time with something kind of sensible … and maybe I wouldn’t have done that if I had a husband that I could do more things together with. But this is the way it is now. Interview 9

Some spouses (four to six) said the wanted to protect their children from seeing the decline of the parent with young onset dementia, and they therefore avoided talking about the severity of the situation with the children. They mentioned several causes for this avoidance, one of which was the genetic aspect to the illness. The spouse did not want their children to see what they might have to go through themselves if they were to develop the illness. One spouse also mentioned that they did not want to scare away the children’s significant others, thinking that the disease might cause them to end relationship.

Yes, keep up some sort of façade. Maybe some sort of façade for them not to feel bad that they might have to walk the same path, or think if their partners see it and thinks, ‘oh my God I don’t want to be a part of this’, and leaves. Interview 4

Some spouses (four to six) also said that they did not want to share difficulties with the children because they didn’t want to change the parent-child relationship.

#### Finding ways to recharge

A source of recharge was for some of the spouses (four to six) their place of work, it was where they could focus on things other than the needs of the person with young onset dementia. They were able to socialize with their co-workers and “recharge their batteries”. The importance of work was also expressed when the spouses spoke of having something to do when the time came for the person with young onset dementia to no longer be living with them. Other spouses spoke of a need to be home alone, a need which they had not felt before.

… before I had gone with him as well. So that, but today I would have told him that no you can go alone so that I can get some me-time, just because right now we are together so much. Interview 6

Some spouses (four to six) said that exercise had become a thing that was neither work nor home, but something just for themselves, and therefore a valuable source for recharging. They described that during the pandemic, extracurricular activities were even more crucial for recharging because they did not have co-workers to socialize with.

But I golf a lot, so, I meet-up with them [golfing buddies] and we travel around as well. I meet quite a lot of people that I get to talk to and so on. That’s very valuable. Interview 5

### Calling for support

The spouses described relying on support from family and friends and co-workers more than health care providers. The nature of support from those sources varied, but all agreed that support from health care providers left something to be desired.

#### Informal support

Some spouses (four to six) said that they had open communication with their extended family, while other had poor communication. Some spouses (four to six) had support from their own or their spouse’s parents, while others had none. Some spouses (four to six) felt they had support from friends, while others felt that they had lost many friends due to their spouse’s illness. Another experience was that of friends not being able to see the inabilities of the person with young onset dementia, because those problems were too subtle.

No one thinks it’s that serious with my husband. They are just like “no we can’t tell that there is anything wrong.” So, you just don’t talk to them about it. Interview 3

Some of the spouses (four to six) said that they wanted to be transparent with co-workers in the interests of understanding if they ever needed to take time off work to support the person with young onset dementia. A few spouses (three) said they had the support of co-workers while others didn’t have that experience. One of the spouses had more solitary positions at work and therefore didn’t have close co-workers.

For the spouses that had children, they described that they could have used the support of the person with young onset dementia in areas that concerned their children. Even though their children were adults and no longer resided at home, difficult situations could still arise that would have preferably been handled by both spouses.

You have a lot to discuss as parents when you have a child together. Even though our daughter was grown and very independent, there was a lot going on with her back then. And I had nobody. I had no one to talk to about it. It has been very difficult. Interview 3

#### Formal support

Some of the spouses (four to six) said that there was insufficient support for the person with young onset dementia. They said that focus had been on receiving a diagnosis for the sick leave application and symptom-relieving medications.

But, I mean don’t they know anything? Don’t they have any other ambition than to medicate? The doctor was like, ‘well you have your medication and your sick leave what else do you want?’ Interview 4

The experience of most spouses (seven to eight) was that the information given to them about dementia was not applicable to people with young onset dementia rather it was aimed at older people with dementia. They said that they could have used some practical help, like getting a checklist of sorts to keep track of what needs to be done, and what could be expected at different stages of the illness. The spouses said that they had requested support targeted at them as a couple rather than individually; however, this service was not available through the memory clinic. The spouses also expressed disappointment that the person with young onset dementia hadn’t received more support.

… maybe it’s like throwing your money away … I don’t know if that’s what they think that there’s nothing to be done. But if you get some kind of serious illness, I thought that health care providers would have offered some sort of support. Interview 4

Spouses who attended support groups thought that it was too difficult to hear about other persons with young onset dementia who were further along in their illness as well as the participants being older. The meetings only left them feeling sad and worried about what was to come. One spouse felt that the problems were too minor to be addressed in a support group. During the pandemic, some of the support groups had met online, which the spouses said made it difficult to connect with others in the same situation. Some spouses said they had not been offered anything other than the support group setting.

No, I could have used someone to talk to, I really think so. Eh, could have, and like I said, I find it a bit strange that no one [in health care] has ever asked me if I need help in all of this. Interview 9

However, one spouse expressed receiving one on one therapeutic conversations and found it helpful. Also, most of the spouses expressed having received adequate financial support from the social insurance agency.

## Discussion

The aim of the study was to explore spouses’ experiences of everyday life when living with a person with young onset dementia. The results showed spouses *Experiencing an emotional impact*, *Using coping strategies*, and *Calling for support*.

A common experience of spouses of persons with young onset dementia is loneliness stemming from not recognizing the person. Spouses in this study were no exception, mentioning that they didn’t get the same response in conversations as before. Previous research has shown that family caregivers of persons with young onset dementia felt gradual loss and were unable to engage in the relationship (Lindauer & Harvath, 2014; Millenaar et al., 2016; Wawrziczny et al., 2016) and felt feelings of alienation (Pang & Lee, 2019; Wawrziczny et al., 2016). Other research found that communication dwindled as the illness progressed, and for some, communication could be completely absent (Larochette et al., 2020; Pang & Lee, 2019). Even though similar experiences of loneliness have been stated for spouses of people with late onset dementia (Greenwood et al., 2019; Leggett et al., 2020) the support available is not as suitable for spouses and people with young onset dementia (Cations et al., 2017). Additional research to increase knowledge about communication as a dyad with a person with young onset dementia would be valuable because such knowledge might help a spouse feel less lonely and that they have a better understanding of the different stages of the illness. This study showed that there was a desire to receive support as a dyad and as an individual. A previous study showed that spouses needed support with managing stressors in everyday life, such as identity changes in the person with early-onset dementia (Grunberg et al., 2022). Such support has been shown to help spouses resume communication that had been lost and can help to accept and understand personality changes due to the illness (Larochette et al., 2020). A study by Williams et al. (2017) has shown that a psychoeducational intervention can improve communication between people with dementia and their family caregivers, mainly amongst people in the early stages of dementia. Furthermore, there is a communication tool measuring both verbal and non-verbal communication of persons with dementia (VNVIS-CR scale) which has been shown to be helpful (Williams et al., 2018). Difficulty communicating has also been found between family carers and healthcare providers when it came to care of people in the later stages of dementia (Anantapong et al., 2022). Other research showed that support early on after diagnosis could decrease stress for both the person with young onset dementia and their family caregiver (Bannon et al., 2020). Providing communication tools or offering support individually, as well as a dyad, could aid spousal coping in everyday life.

Spouses of persons with young onset dementia experience a wide range of emotions. One frequently experienced emotion was frustration. The emotions experienced can be explained by the equity theory (Hatfield et al., 1979, a relationship that is unbalanced when it comes to responsibilities both inside and outside the home will cause the disadvantaged person in the relationship to feel angry and distressed. The spouses in the current study expressed feeling worried and anxious about the future. In this study, spouses found that having conversations about the future caused feelings of anxiety, and so they therefore preferred to focus on the present. Previous research has found similar behaviours, and that spouses of persons with young onset dementia rather preferred to reminisce about the past than to address the future (Pang & Lee, 2019; Spreadbury & Kipps, 2019; Wawrziczny et al., 2016). Hoppe (2019) stated that families and people with young onset dementia are impacted differently due to them being at a stage in life where they have more family obligations as well as financial obligations than those with late onset dementia. A previous study showed that spouses expressed a need for support to help them plan. Spouses felt better prepared to handle what was to come after receiving additional information, which included nursing home planning, medical information, and information about financial and legal concerns (Grunberg et al., 2022). Other research found that getting support as a dyad improved communication (Bannon et al., 2020). Further research is needed to get a better understanding of how to help spouses have important conversations regarding the future.

Spouses of persons with young onset dementia are resourceful when coping with everyday life. This study showed that the spouses of persons with young onset dementia were creative in finding practical ways to deal with everyday life. Previous research has also shown that caregivers of persons with young onset dementia find ways to cope, such as lowering expectations of the person with young onset dementia, and finding ways to recharge (Spreadbury & Kipps, 2019). Other research has documented the resourceful ways found by family caregivers to cope with the situation, such as adopting positive communication and having a teamwork approach to solving problems (Bannon et al., 2022). It could be argued that this resourcefulness is somewhat forced to happen since the aimed support is inadequate for this group. Kilty et al. (2023) describe how family caregivers and people with young onset dementia has had to fight for support due to them being under the age of 65. Further research is need for a more thorough understanding of coping abilities.

Kimura et al. (2019) showed that family caregivers of people with early-onset dementia who themselves had moderate to high resilience abilities had lower levels of depression and anxiety (the level of resilience was unrelated to whether the family caregiver received assistance or not). Resilience is defined as “the process and outcome of successfully adapting to difficult or challenging life experiences, especially through mental, emotional, and behavioral flexibility, and adjustment to external and internal demands” and it is a skill that can be improved through practice (American Psychological Association, n.d.). Previous research identified attributes of resilience amongst family caregivers of persons with young onset dementia, such as resourcefulness, social support, self-efficacy, flexibility, positive thinking, and spirituality (Kobiske & Bekhet, 2018). Strengthening resilience might be important for spouses of persons with young onset dementia so that they can lower their mental distress, and further research on what affects resilience abilities of spouses of persons with young onset dementia is needed. However, using equity theory it is easy to understand the spouses at times feeling frustrated and anger.

Support groups for spouses living with a person with young onset dementia are not always valuable. This study showed that when the spouses listened to other spouses’ experiences of living with a person dementia, especially if that person was further along in their illness or living with a person with late onset dementia, it caused anxiety and they felt out of place rather than being helpful. A previous study showed that spouses of persons with young onset dementia felt disconnected when placed in a group with older members, or members with spouses who were further along their illness (Grunberg et al., 2022). Spouses of persons with young onset dementia have also felt stressed by feelings that they were outsiders with little support available to them (Kaiser & Panegyres, 2007). It is important that support groups are divided by the specific situation of the spouses, to make them feel a part of a group that understands their situation.

### Strengths and limitations

The research shows trustworthiness (Lincoln & Guba, 1985) in the following ways. The nine spouses in this study were diverse, including both men and women residing in rural and urban areas. Healthcare services provided where from five different memory clinics and municipals. Interviews were not held in the presence of the person with young onset dementia so that the spouses would be able to express their experiences freely. Although there were only nine interviews, the data collected was rich in content and lengthy.

The ages, genders, sexual orientation and educational levels of the spouses varied, giving the data credibility. The data was analysed systematically through a qualitative content analysis (Granheim & Lundman, 2004; Granheim et al., 2017), a well proven and suitable method to analyse qualitative data. The first author has nursing experience working with people with dementia and their spouses and was aware of the pre-understanding which also was discussed it in the research group. By being made aware of one’s own pre-understanding prior to analysing the data, the pre-understanding could be placed in the background throughout the analysing process. The results also have been strengthened with several quotes from the participants to further support that it was the experience of the participants. This also to show neutrality of the researcher and the results (Polit & Beck, 2018). The focus was on spouses expressing their experience of living with a person with young onset dementia. The analysis was discussed at length within the author group on several occasions to ensure relevance and accuracy. The resulting themes and sub-themes were created through consensus. The interviews were conducted during the COVID −19 pandemic, which could be a limitation in terms of dependability. Because the spouses were all under the age of 65, their experiences might not be transferable to older spouses. The spouses in the current study might have commitments that older spouses might not, and they might therefore perceive situations differently. However, the clear description in both text as well as in Table 1, where the characteristics of the participants and the context is well depicted enhances transferability (Granheim & Lundman, 2004). A criterion for exclusion was an inability to speak Swedish, and the perspectives of non-Swedish speakers were therefore lost. Having excluded that group could limit transferability and result in a skewed view of everyday life. The spouses in this study were all middle class which could have affected the results, as financial worries were close to nonexistent. Sweden’s welfare system is unlike most other countries, which also could have contributed to the spouses not expressing financial concerns. This could therefore limit the transferability to spouses living in other countries with little or no welfare.

The first author has worked as a psychiatric nurse in an inpatient care unit and has been in contact with persons with dementia and their spouses. This prior experience was with those at a much later stage of the illness who were no longer residing at home. However, a pre-understanding of the illness, and what it leads to might have affected the interviews, as well as interpretation of the data. Analysis of the data involved all the authors in the research group to ensure credibility. The result of the current study is supported by previous research, which strengthens the dependability of the results.

## Conclusion

The spouses in this study expressed feeling a wide range of emotions living with a person with young onset dementia. The different emotions can be understood by the equity theory which explains that emotions arise due to the imbalance in the relationship. The spouses experienced something lacking regarding support from healthcare providers. The support that was available was in many cases not aimed at spouses of people with young onset dementia and in the early stages of the illness. The spouses had found ways to recharge their own physical and mental health so that they could manage added responsibilities at home, whilst juggling work. This proposes a change in the support being provided to better suit the spouse’s needs. The spouses experienced both feeling supported by friends but also alienation by family and friends. This indicates that there needs to be an increased understanding in society regarding people's preconceived notions regarding dementia. The spouses also expressed needing the understanding of their employer as well as co-workers, should they have to take time off work to support the person with dementia or accompany to doctor visits. With an elevated retirement age and the risk of being diagnosed with dementia increasing with age it could be an indication of additional spouses of people with dementia in the workplace in the future. This proposes a need for increased knowledge regarding the needs of spouses of people with young onset dementia as well as added research regarding people with dementia in the workplace.

## Supplementary Material

Appendix.docx

## Appendix

**Themes and subthemes with underlying content** Note: The numbers 1-9 refer to spouse nr 1, spouse nr 2 and so on.

**Experiencing an emotional impact**

**Loneliness in a dyad**

Stopped consulting (2, 3, 4, 5, 6, 9)

Not on the same wavelength (2, 3, 4, 5, 6, 9)

Repetitive conversations (1, 2, 3, 4, 5, 6, 7, 9)

Feeling that the person is unempathetic (2, 3, 4)

Feeling estrangement (2, 3, 4, 5, 9)

Left alone with problems (2, 3, 4, 5, 6, 9)

Feeling hopeless (2, 3, 9)

Loss of travelling (2, 3, 5, 8, 9)

Unrealistic view (4, 5, 8, 9)

**Feelings of frustration**

Changed behaviour/inabilities (2, 3, 4, 5, 6, 8, 9)

Not understanding the illness (6, 7, 9)

Frustration at oneself (2, 3, 4, 6

Not the same output (2, 3, 4, 5, 6, 9)

No alone time (2, 3, 6, 9)

Not being able to rely on digital aids (3, 5, 9)

Having to help/take over responsibilities due to inabilities (2, 3, 4, 5, 6, 7, 9)

Not being independent anymore (2, 3, 4, 5, 6, 9)

Maintaining social contacts (2, 3, 4, 5, 6, 9)

Sole parental responsibility (2, 3, 5, 9)

**Increasingly worried**

Getting lost while out (5, 6)

Heredity of the illness (4, 5, 9)

Getting papers in order (1, 5, 6, 8)

Own mortality (1)

Timely support (4, 5, 9)

**Peace of mind**

Meaningful everyday for person with dementia (2, 3, 4, 5, 6, 8, 9)

Having had difficult conversations (4, 8)

**Using coping strategies**

**Adopting a positive mindset**

Focusing on what works (1, 4, 5, 6, 7, 8)

Alzheimer in the family (4, 7, 8)

Seizing the day (1, 6, 8)

Not minding added responsibility (1, 7, 8)

**Adapting to inabilities**

Avoiding stress (1, 2, 3, 6, 7, 8, 9)

Avoiding arguments (1, 2, 3, 4, 5, 6, 7, 8, 9)

Not wanting to intrude on integrity (1, 4, 5, 6, 7, 8, 9)

Clues in conversations (1, 6, 7, 8)

Leaving time for power naps (3, 6, 8)

**Adopting an avoidant approach**

Difficult conversations (1, 5, 6, 7, 9)

Avoiding home (2, 3, 4, 5, 9)

Protecting the children (3, 4, 5, 9)

**Finding ways to recharge**

Work (2, 4, 5, 6, 7)

Extracurricular activities (2, 3, 4, 5, 8, 9)

**Calling for support**

**Informal support**

Open/poor communication with extended family (2, 3, 4, 5, 6, 7, 9)

Support/inadequate support from friends (2, 3, 4, 5, 6, 7, 8, 9)

Support/inadequate support from co-workers (2, 4, 5, 6)

Inadequate support from the person with dementia (2, 3, 4, 5, 9)

**Formal support**

Inadequate support for the person with dementia (2, 4, 5, 8, 9)

In need of checklists, aimed information etc. (1, 4, 5, 6, 7, 8, 9)

Adequate support from healthcare (3)

Inadequate support from healthcare (1, 2, 4, 5, 6, 7, 8, 9)

Adequate support from social security agency (1, 2, 3, 4, 5, 6, 8, 9)
