# Supplementary material for: Living with a person with young onset dementia – spousal experience
Source: Int J Qual Stud Health Well-being. 2024 Mar 17;19(1):2330233. doi: 10.1080/17482631.2024.2330233 (PMC10946261; doi:10.1080/17482631.2024.2330233)
Supplement: Appendix.docx [file ZQHW_A_2330233_SM2239.docx]

**Appendix**

**Themes and subthemes with underlying content** Note: The numbers 1-9 refer to spouse nr 1, spouse nr 2 and so on.

**Experiencing an emotional impact**

**Loneliness in a dyad**

Stopped consulting (2, 3, 4, 5, 6, 9)

Not on the same wavelength (2, 3, 4, 5, 6, 9)

Repetitive conversations (1, 2, 3, 4, 5, 6, 7, 9)

Feeling that the person is unempathetic (2, 3, 4)

Feeling estrangement (2, 3, 4, 5, 9)

Left alone with problems (2, 3, 4, 5, 6, 9)

Feeling hopeless (2, 3, 9)

Loss of traveling (2, 3, 5, 8, 9)

Unrealistic view (4, 5, 8, 9)

**Feelings of frustration**

Changed behavior/inabilities (2, 3, 4, 5, 6, 8, 9)

Not understanding the illness (6, 7, 9)

Frustration at oneself (2, 3, 4, 6

Not the same output (2, 3, 4, 5, 6, 9)

No alone time (2, 3, 6, 9)

Not being able to rely on digital aids (3, 5, 9)

Having to help/take over responsibilities due to inabilities (2, 3, 4, 5, 6, 7, 9)

Not being independent anymore (2, 3, 4, 5, 6, 9)

Maintaining social contacts (2, 3, 4, 5, 6, 9)

Sole parental responsibility (2, 3, 5, 9)

**Increasingly worried**

Getting lost while out (5, 6)

Heredity of the illness (4, 5, 9)

Getting papers in order (1, 5, 6, 8)

Own mortality (1)

Timely support (4, 5, 9)

**Peace of mind**

Meaningful everyday for person with dementia (2, 3, 4, 5, 6, 8, 9)

Having had difficult conversations (4, 8)

**Using coping strategies**

**Adopting a positive mindset**

Focusing on what works (1, 4, 5, 6, 7, 8)

Alzheimer in the family (4, 7, 8)

Seizing the day (1, 6, 8)

Not minding added responsibility (1, 7, 8)

**Adapting to inabilities**

Avoiding stress (1, 2, 3, 6, 7, 8, 9)

Avoiding arguments (1, 2, 3, 4, 5, 6, 7, 8, 9)

Not wanting to intrude on integrity (1, 4, 5, 6, 7, 8, 9)

Clues in conversations (1, 6, 7, 8)

Leaving time for powernaps (3, 6, 8)

**Adopting an avoidant approach**

Difficult conversations (1, 5, 6, 7, 9)

Avoiding home (2, 3, 4, 5, 9)

Protecting the children (3, 4, 5, 9)

**Finding ways to recharge**

Work (2, 4, 5, 6, 7)

Extracurricular activities (2, 3, 4, 5, 8, 9)

**Calling for support**

**Informal support**

Open/poor communication with extended family (2, 3, 4, 5, 6, 7, 9)

Support/inadequate support from friends (2, 3, 4, 5, 6, 7, 8, 9)

Support/inadequate support from co-workers (2, 4, 5, 6)

Inadequate support from the person with dementia (2, 3, 4, 5, 9)

**Formal support**

Inadequate support for the person with dementia (2, 4, 5, 8, 9)

In need of checklists, aimed information etc. (1, 4, 5, 6, 7, 8, 9)

Adequate support from healthcare (3)

Inadequate support from healthcare (1, 2, 4, 5, 6, 7, 8, 9)

Adequate support from social security agency (1, 2, 3, 4, 5, 6, 8, 9)
